# Association of diminished expression of RASSF1A with promoter methylation in primary gastric cancer from patients of central China

**DOI:** 10.1186/1471-2407-7-120

**Published:** 2007-07-03

**Authors:** Mei Ye, Bing Xia, Qiusha Guo, Feng Zhou, Xiaolian Zhang

**Affiliations:** 1 Department of Internal Medicine & Geriatrics, Research Centre of Digestive Diseases, Zhongnan Hospital, Wuhan University, PR China; 2 Key Laboratory of Allergy and Immune-related Diseases, Wuhan University School of Medicine, Wuhan 430071, Hubei Province, PR China

## Abstract

**Background:**

Although methylation-mediated inactivation of expression of RASSF1A, a candidate tumor suppressor gene, has been observed in several human cancers, the data concerning alteration of RASSF1A expression and methylation in Chinese primary gastric cancer are scarce. Moreover, direct evidence showing the association between protein expression of RASSF1A and primary human cancers is lacking. The aim of this study was to investigate RASSF1A expression in tissue of primary gastric cancer (GC) at mRNA and protein levels, and to establish the possible relationship between DNA methylation status and protein expression of RASSF1A in Chinese.

**Methods:**

Fifty-four patients with primary gastric cancers were included in the study of RASSF1A mRNA expression and methylation status between the cancer tissue and the corresponding adjacent normal tissue. 20 out of 54 patients were included for study of RASSF1A protein expression. The expression of RASSF1A at mRNA and protein levels was determined by RT-PCR and Western-blotting, respectively. The RASSF1A promoter methylation was detected by methylation-specific PCR.

**Results:**

RASSF1A mRNA and protein expressions in GC were reduced significantly with comparison to the corresponding normal tissues (OD value: 0.2589 ± 0.2407 vs 0.5448 ± 0.2971, *P *< 0.0001; 0.1874 ± 0.0737 vs 0.6654 ± 0.2201, *P *< 0.0001, respectively). Methylation frequency of RASSF1A in primary GC is higher than that in the corresponding normal tissues (66.7% vs. 14.8%, *P *< 0.0001). Furthermore, RASSF1A mRNA expression in methylation group of GC was further reduced when compared to the unmethylation group of GC (0.1384 ± 0.1142 vs. 0.5018 ± 0.2463, *P *< 0.0001).

**Conclusion:**

Expression of RASSF1A was reduced in tissue of GC at mRNA and protein levels. Diminished expression of RASSF1A was associated with the promoter methylation.

## Background

Gastric cancer (GC) is one of the most common malignancies in gastrointestinal diseases. Although incidence of GC is declining in the developed counties, it is still a leading cause of death from cancers in most developing countries. Both genetic and environmental factors including *H. pylori *infection have been considered to contribute to the development of GC. Recently, several studies have shown that silence of tumor suppressor genes by epigenetic modification is a fundamental mechanism for inactivation of cancer-related genes in the pathogenesis of human cancer [1]. Of the particular note is the fact that promoter hypermethylation plays an essential role in the loss of function of tumor suppressor genes.

Allelic loss at chromosome 3p21.3 is one of the most frequent genetic changes in various types of human cancers [2]. In the late 90's, Dammann [3] and Burbee [4] et al identified RASSF1A, a novel gene from the common homozygous deletion area at 3p21.3. This gene shares homology with mammalian RAS effectors. There are seven RASSF1 isoforms (A to G) generated through alternative splicing RASSF1A has been shown to reduce tumorigenicity in vivo and is frequently inactivated in a variety of primary human cancers. Genetic mutations in this isoform are rare. It is known that epigenetic silencing of RASSF1A associates with CpG islands methylation of the promoter region. Bisulfate DNA sequencing has demonstrated that hypermethylation of promoter region inactivates RASSF1A expression in the major human cancers. This is supported by the observation of re-expression of RASSF1A in various cancer cell lines treated with demethylating agents [4-7]. To date, several studies have shown the presence of aberrant promoter methylation of RASSF1A and loss or abnormal low expression of RASSF1A in primary GC, suggesting that inactivation of RASSF1A expression is closely associated with the promoter methylation [5,8-10]. To our best knowledge, no data concerning the expression of RASSF1A and methylation status in GC in Chinese population are available. In the present study, we have determined expression of RASSF1A at both mRNA and protein levels, and their association with RASSF1A promote methylation status in Chinese primary GC in the central China.

## Methods

### Patients

Fifty-four patients with primary GC were registered in the Wuhan University Zhongnan Hospital and the Hubei Provincial Cancer Hospital from December 2003 to June 2005, and all of the patients underwent curative resection with systematic lymph node dissection. GC tissues and corresponding adjacent normal gastric tissues were obtained from operations before chemotherapy. The patients included 35 males and 19 females, mean age 57.6 ± 13.7 years (range from 30 to 85 years). Diagnosis and differentiations of GC were determined by histopathological examinations and the TNM classification. The demographic and clinicopathological data of the patients are summarized in Table 1. Among 54 patients with GC, 20 cases were included for study of protein expression of RASSF1A. The protocol of the study was approved by the ethic committee of Wuhan University Zhongnan Hospital. Tumor and adjacent normal tissues were obtained at the time of surgery, snap-frozen in liquid N_2 _and stored at -80°C until used. Sample sections were stained in H&E and were examined by two experienced pathologists.

**Table 1:** Association of RASSF1A gene methylation with demographics and clinicopathological features of primary gastric cancer (n = 54)

	*Methylation*		*P value*	*OR (95%CI)*
			
	Yes	No		
Gender				
male	24	11	0.766	1.273 (0.393~4.117)
female	12	7		
Age				
< 60	17	13	0.145	0.344 (0.101~1.167)
≥ 60	19	5		
Tumor sites				
antrum	14	7		
body and cardiac	22	11	1.000	1.000 (0.313~3.192)
Stages				
early	1	2	0.255	0.229 (0.019~2.708)
advanced	35	16		
Lymph node metastasis				
yes	22	7	0.154	2.469 (0.774~7.882)
no	14	11		
Distant metastasis				
yes	5	1	0.651	2.742 (0.296~25.424)
no	31	17		
Differentiations				
well and moderate	18	12	0.384	0.500 (0.154~1.624)
poor	18	6		
Lauren's				
Intestinal type	12	10	0.148	0.400(0.125~1.275)
Diffuse type	24	8		

### DNA and RNA extract

DNA from frozen tissues was purified by phenol/chloroform and ethanol precipitation and dissolved in 50 μl of distilled water and stored at -20°C until use. Total RNA was isolated using the Trizol kits (Molecular Research Center Inc., Cincinnati, USA) according to the manufacturer's instruction.

### Semi-quantitative RT-PCR

Three micrograms of total RNA were reversely transcribed using RevertAid™ first strand cDNA synthesis kit (Fermentas Inc., Vilnius, Lithuania). All total RNA were treated by DNase to avoid contamination of genomic DNA. The primers for RASSF1A and glyceraldehydes-3-phosphate dehydrogenase (GAPDH) were designed based on RASSF1A gene (GeneBank Accession #: NM_007182) and GAPDH gene (GeneBank Accession #: NM_002046). The primers for RASSF1A were 5'-CTT TTA CCT GCC CAA GGA TGC-3' and 5'-CAC CTC CCC AGA GTC ATT TTC C-3'. The primers for GAPDH (5'-CAT GAC AAC TTT GGT ATC GTG-3', and 5'-GTG TCG CTG TTG AAG TCG TCA GA-3') were used as internal control. The thermal cycles were: 94°C for 5min, followed by 35 cycles of 94°C for 30sec, 55°C for 45sec and 72°C for 60sec, and finally 72°C for 5min for extension. The PCR products were separated in 2% agarose gel in electrophoresis and visualized with ethidium bromide staining, and quantified using an image analysis system (UVP Bioimaing systems, USA). The generated PCR products were 265bp for RASSF1A and 370 bp for GAPDH. Optical density (OD) value of the mRNA expression was calculated.

### Bisulfate modification

DNA from tumor tissues and corresponding adjacent normal tissues were subjected to bisulfate treatment according to Herman et al [11]. Briefly, 2 μg of genomic DNA suspended in 50 μl distilled water, denatured with 0.2 M NaOH for 10 min at 37°C, and added 30 μl of freshly prepared 3 M sodium bisulfate (pH 5.0). The samples were over layered with mineral oil to cover surface of the aqueous phase, and incubated at 50°C for 16~20 h. The DNA sample was desalted through a column of Wizard DNA Clean-up System (Promega, Wisconsin, USA) according to the manufacturer's instructions. The samples were treated with 0.3 M NaOH for 5 min at room temperature and precipitated with ethanol. The bisulfate-modified genomic DNA was re-suspended in 50 μl TE buffer (10 mM Tris·Cl, 1 mM EDTA, pH7.6) and stored at -80°C for use.

### Methylation-specific PCR (MSP)

The methylation status of RASSF1A was determined by MSP. Bisulfate treatment of genomic DNA converts cytosine to uracil bases but has no effect on methylcytosine. The specific PCR was then used to distinguish between methylated and unmethylated DNA sequences in a HotStar TaqE PCR machine (TaKaRa Bio, Co., Ltd., Tokyo, Japan). The primers for the methylated form were 5'-GGG TTT TGC GAG AGC GCG-3' and 5'-GAT AAC AAA CGC GAA CCG-3', and the primers for the unmethylated form were 5'-GGG GTT TTG TGA GAG TGTG-3' and 5'-ACT AAC AAA CAC AAA CCA AAC-3' (Gene Bank Accession: AC002481). The PCR condition consisted of one incubation of 15 min at 95°C, followed by 40 cycles of a 30 sec denaturation at 94°C, 50 sec at an annealing temperature (64°C for methylated and 59°C for unmethylated specific primers), and a 30 sec extension at 72°C, and a final extension at 72°C for 10 min. PCR products were separated in 8% polyacrylamide gels in electrophoresis. Genomic DNA, methylated in vitro by CpG methyltransferase (Sss I) following the manufacturer's directions (New England BioLabs, Inc., Beverly, MA), was used as a positive control. Water blank was used as a negative control. Each primer set generated a 169 bp product.

### Western blotting

Fifty mg samples were homogenized and centrifuged at 12,000–15,000 g for 10–15 min in ice-cold 50 mM HEPES buffer (pH 7.4) containing 150 mM NaCl, 50 mM Tris·Cl (pH 8.0), 1% Triton X-100, 2 mM EDTA, 2 mM PMSF, 5 μg/ml leupeptin, 1% NP-40, and 10% glycerol. Supernate was collected and protein concentration was then measured using BSA as a standard by the BCA Protein Assay Reagent (Pierce Biotechnology., Inc., Rockford, USA). Equal amounts (100 μg) of total protein from tissues were electrophoresed on SDS-polyacrylamide gels (10% gel) and transferred to polyvinylidine difluoride (PVDF) filter membranes for 30 min bysemi-dry blotting. The membranes were blocked for 2 h at room temperature with the block solution provided in the ECL kit (Protein Detector LumiGLO Reserve™ Western Blotting Kit, KPL, Maryland, USA), and then incubated for 45 min with a 1:100 dilution of the primary antibody to RASSF1A. The primary antibody used for Western blot analysis was an affinity purified goat polyclonal antibody to RASSF1A, C-12 (SC-18724, Santa Cruz Biotechnology Inc., Santa Cruz, Calif., USA). The PVDF membranes were then washed for 30 min using a wash solution, followed by an 1 h incubation with anti-goat antibody conjugated to horseradish peroxidase in block solution (Rabbit anti-goat IgG, Chemicon Internation, Inc., Temecula, California, USA). ThePVDF membranes were washed again and the immunoreactive bands were detected by enhanced chemiluminescence and visualized by image analysis system (Bio-Rad Laboratories, Inc. Hercules, CA, USA). GAPDH (Abcam Biotechnology, Cambridge, UK) was used as control.

### Statistical analysis

Statistical analyses were performed using the SPSS11.5 software (SPSS, Inc., Chicago, IL). The differences between the variables were assessed by chi square test or t-tests according to the data. The statistical significance of differences was set as *P *< 0.05.

## Results

### RASSF1A mRNA expression in gastric cancer

**Figure 1:**
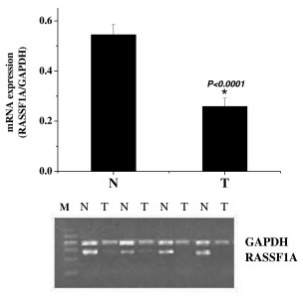
Expression of RAASF1A mRNA in primary gastric cancer (T) and corresponding normal tissue (N). RT-PCR products for RASSF1A were quantified by densitometric scanning of in ethidium bromide-stained gels compared with GAPDH.

RASSF1A mRNA expression in 54 GC tissues was 50% of that in the adjacent corresponding normal tissues, which revealed mRNA expression in tumors was significantly reduced compared with that of the normal tissues as shown in Fig. 1.

### RASSF1A protein expression in gastric cancer

**Figure 2:**
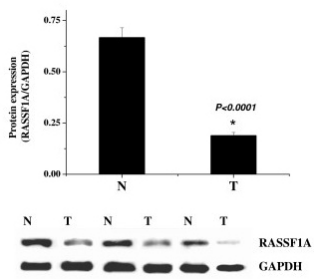
Protein expression of RASSF1A in primary gastric cancer (T) and corresponding normal tissue (N). Protein level of RASSF1A was quantified by Western-blotting compared with GAPDH.

Consistent to the decrease in mRNA expression, figure 2 shows that the protein expression in tumors was only 21.4% of that in normal tissues, which suggested that the protein level of RASSF1A expression was also reduced with comparison to the corresponding normal tissues.

### RASSF1A mRNA expression and DNA methylation in promoter of RASSF1A gene in primary gastric cancer

**Figure 3:**
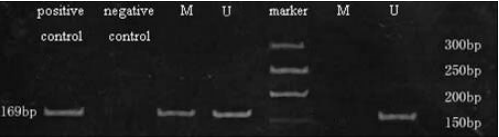
Methylation of RASSFIA in primary gastric cancer and corresponding normal tissue by MSP. Lane 3, 4 and lane 6, 7 show a representative result from a tumor and normal sample, respectively. U: amplified product with primer recognizing unmethylated sequences; M: amplified product with primer recognizing methylated sequences. Genomic DNA, methylated in vitro by CpG methylase (Sss I) was used as a positive control. Water blank was used as a negative control. The PCR products were resolved on a 8% polyacrylamide gels

As shown in Fig. 3, methylation frequency of RASSF1A in GC was 3.5-fold higher than that in the corresponding normal tissues (66.7% vs 14.8%, P < 0.0001). RASSF1A mRNA expression between methylation and unmethylation groups in GC were significantly different, the former is 2.6-fold lower than the latter (0.1384 ± 0.1142 vs 0.5018 ± 0.2463, *P *< 0.0001). The results showed that hypermethylation at the CpG island in the RASSF1A promoter is strongly associated with significantly reduced mRNA expression of RASSF1A.

### Association between methylation and clinicopathological parameters of gastric cancer

The association between promoter methylation and relevant demographic and clinicopathological characteristics including gender, age, tumor site, lymph node metastasis, distant metastasis, differentiation, stage and Lauren's type were shown in Table 1. There was no significant association between promoter methylation and the clinicopathological parameters.

## Discussion

In the present study we provided evidence that RASSF1A expression in GC was reduced at mRNA and protein levels and the methylation in promoter region of the RASSF1A gene was 3.5-fold more frequent in primary GC than in corresponding normal tissues. In addition, the incidence of GC patients with RASSF1A promoter methylated over unmethylated was 2-fold higher. These suggest that RASSF1A expression in GC was highly associated with the promoter methylation status of the RASSF1A gene. To further confirm the relationship between expression of RASSF1A and the methylation of the promoter, we compared RASSF1A mRNA expression between methylation and unmethylation tissues in GC, and found that aberrant methylation of the RASSF1A gene promoter region is responsible for the reduction or loss of RASSF1A mRNA expression in primary GC. Our study was consistent with one study which showed that 45.6% primary GC had none or abnormally low mRNA expression of RASSF1A [5]. Among 30 matched sets from the same patients 73.3% of GC was found to have reduced RASSF1A expression [5]. To our best knowledge, no study of RASSF1A protein expression in GC has been reported. We further analyzed RASSF1A expression at protein level by Western blotting in 20 GC patients and found that the protein expression in tumors is only 21.4% of the expression in normal tissues. This result indicated that RASSF1A protein expression in GC was significantly lower than in corresponding adjacent normal tissues. This is consistent to the mRNA expression of RASSF1A.

The mechanism of reduced or silenced RASSF1A expression could be caused by promoter methylation and/or mutation of RASSF1A gene. However, several studies have shown that mutation of RASSF1A gene is very rare [4,5,7,12,13]. Thus, RASSF1A gene is probably inactivated by mechanisms of methyltion. Byun et al [5] found that of 41 primary GC with loss or abnormal reduction of RASSF1A expression, 39 were methylated, whereas in both GC and normal tissues with normal RASSF1A expression none were methylated. To et al[10] showed that hypermethylation of RASSF1A promoter was found in 25.8% of GC and in 11.1% of the gastric intestinal metaplasia(IM), respectively. Our data has shown that the frequency of RASSF1A methylation in GC was higher than the above reported data. GC is highly prevalent in China and high methylation of RASSF1A may explain partly the reason for why GC incidence is higher in the Chinese. Interestingly, the fact that RASSF1A promoter hypermethylation is higher in EBV positive GC than in EBV negative carcinoma (66.7% vs 3.6%) may suggest a close association between EBV and aberrant methylation in GC. Indeed, a previous report has indicated that viral oncogenesis might involve aberrant methylation, resulting in inactivation of tumor suppressor genes [9].

We found that the methylation frequency of RASSF1A in primary GC and in the corresponding normal tissues were 66.7% and 14.8%, respectively. Our data showed that out of 54 GC patients 36 were methylated and 18 were unmethylated, suggesting methylated GC is more common, in agreement with the other reports. The previous study [14] suggested that the stomach is one of the normal tissues with a high frequency of aging-related methylation, and gastric cancer is one of the tumors with a high frequency of CpG island methylation. Kang et al [15] studied the methylation status of 11 genes in nonneoplastic gastric mucosa and they found that aberrant CpG island methylation occurred frequently in chronic gastritis, which showed aging relationship, and that aging-related methylation was gene type-specific. RASSF1A gene was rarely methylated, with no aging relationship. Furthermore, other authors reported [3-5] that in normal tissues, no methylated RASSF1A alleles were detected. However, our data showed that in the corresponding normal tissues, the RASSF1A methylation frenquency was high percentage (14.8%). The discrepancy between our data and other authors' may be due to the difference of the samples, which we studied were the non-nesoplasia gastric tissues in patients with gastric cancer and it's very likely to include chronic gastritis tissues, especially intestinal metaplasia tissues in the corresponding adjacent normal tissues. The previous study reported that several genes were frequently methylated in chronic gastritis, especially in intestinal metaplasia tissues. To et al [10] examined the 8 tumor-related gene methylation status including RASSF1A in gastric intestinal metaplasia in patients with and without gastric cancer, and they found that the frequency of methylation in RASSF1A in cancer and intestinal metaplasia were 25.8% and 11.1%, respectively. The result was consistent with ours. In fact, we sequenced the MSP products for several samples. The sequence was consistent to the sequence predicted (data not show). We found that unmethylated bands always coexist with methylated bands in most primary GC and corresponding adjacent normal tissues in spite of reduction or loss of RASSF1A expression, consistent with previous reports [4,10]. This is most likely these resected tumors were not microdissected and were contaminated with stromal cells. Additionally, MSP is extremely sensitive. Bai et al [16] thought that detection of these two bands represents either intra-allelic heterogeneity of the CpG island methylation or methylation of the island varying from allele to allele. Alternatively, our result might indicate that partial methylation of the RASSF1A gene promoter is enough to silence the gene transcription since loss of a single gene copy is sufficient to promote tumor formation [17,18]. Tommasi et al [19] observed that heterozygous RASSF1A knockout mice were significantly tumor-prone, both for spontaneous tumor formation and for the chemically induced tumors. This may suggest that the RASSF1A gene has the characteristics of conferring haploinsufficiency when only one allele is lost.

We also investigate the association between promoter hypermethylation and relevant clinicopathologic parameters including age and gender of the patients, site of carcinoma, histological differentiation, Lauren's type and metastasis of GC. We found that frequency of the methylation of RASSF1A gene was markedly higher in advanced tumors compared with early stage tumors(68.6% vs 33.3%), and higher in poorly differentiated tumors (75% vs 60%) when compared with well and moderately differentiated tumors. However, these differences don't reach statistical significance. Byun et al [5] have shown that inactivation of RASSF1A was correlated with tumor stage and grade but not with histological types of tumors. The discrepancy may explain by limited samples for early GC in our study.

Transfection of RASSF1A gene reduces the growth of human cancer cell in vitro and in vivo [3,4], supporting a role for RASSF1A as a tumor suppressor gene. RASSF1A activating RAS may require heterodimerization of RASSF1A and the novel Ras effector (NORE1), and induce apoptosis through its interaction with Ras, NORE1, the connector enhancer of KSR (CNK) and the pro-apoptotic MST1 kinase [20]. Several groups have reported that RASSF1A is a microtubule-binding protein and regulates mitotic progression [21-25], and may function in signal transduction pathways involving RAS like small GTPase proteins. Tommasi et al[19] has demonstrated that *Rassf1a*-/- and *Rassf1a +/- *mice have increased tumor multiplicity and tumor size, suggesting further the role of tumor suppression of RASSF1A, which may explain its frequent epigenetic inactivation in human tumors.

## Conclusion

In summary, the expression of RASSF1A was markedly reduced to an abnormal level or completely lost in primary GC compared with adjacent normal tissue, and was correlated to hypermethylation of the promoter of the RASSF1A gene. Further work is necessary to elucidate its exact function and interaction with other factors to develop strategies for early diagnosis, prevention and treatment of gastric cancer.

## Competing interests

The author(s) declare that they have no competing interests.

## Authors' contributions

XB supervised the design of the study and experiments and analyzed and interpreted of data. YM conceived of the study, and carried out the Western-blotting and draft the manuscript. GQS carried out the Bisulfate modification and MSP studies. ZF was participated in the register and collection of clinical data and carried out the RT-PCR. ZXL performed the statistical analysis. All authors read and approved the final manuscript.

## Pre-publication history

The pre-publication history for this paper can be accessed here:


